# Regeneration of the lizard heart after cryoinjury

**DOI:** 10.1113/EP093688

**Published:** 2026-05-07

**Authors:** Martina Gregorovicova, Barbora Sankova, Martin Bartos, Bjarke Jensen, Tobias Wang, David Sedmera

**Affiliations:** ^1^ First Faculty of Medicine Institute of Anatomy Charles University Prague Czech Republic; ^2^ Institute of Dental Medicine First Faculty of Medicine Charles University Prague Czech Republic; ^3^ Department of Medical Biology Amsterdam Cardiovascular Sciences University of Amsterdam Amsterdam The Netherlands; ^4^ Department of Zoophysiology Aarhus University Aarhus Denmark

**Keywords:** cardiac, *Eublepharis*, myocardium, reptiles, ventricle

## Abstract

Lizards are renowned for their tremendous potential to heal tissues and organs after injury, but little is known about myocardial regeneration in reptiles generally. Here, we study cardiac regeneration in the leopard gecko (*Eublepharis macularius*) to fill the knowledge gap between traditional models of poikilothermic (zebrafish) and homeothermic (neonatal mice) vertebrates commonly used in myocardial regenerative studies. We inflicted cryoinjury at the apex of the adult heart and used optical mapping, microCT, histology and immunohistochemistry to evaluate myocardial regeneration over the following 4 weeks. Optical mapping revealed epicardial electrical activation of the ventricle during the healing period after cryoinjury, and demonstrated that the cryoinjured area was devoid of electrical activation during the first 2 weeks. The functional recovery of signal spreading over the whole ventricular surface was completed in 21 days after cryoinjury. Morphologically, the first week was characterized by a loss of myocardial viability, the second week by a restoration of the myocardial tissue, the third week by continuation of the regenerative processes, and the fourth week by a completion of myocardial regeneration and a restoration of heart viability. In conclusion, geckos may provide novel insight into myocardial healing across the vertebrate lineages and thus help to reveal healing abilities between non‐ischaemic regenerative and ischaemic reparative processes during cardiac regeneration.

## Abstract (highlights)

- **What is the central question of this study?**
  Does amniotic species such as lizard regenerate after cardiac cryoinjury?
- **What is the main finding and its importance?**
  A model organism, the leopard gecko, heals myocardial damage within 4 weeks and restores ventricular structure and function. Histological analysis suggests the formation of new myocardium, with more cycling nuclei. The leopard gecko provides an amniote model to understand the cardiac regeneration without replacement fibrosis, which is an important finding in context to cardiac healing in mammalian lineage.

## 1 INTRODUCTION

Understanding the ability of animals to regenerate a damaged heart has considerable translational value to improve current treatments of heart failure in response to inadequate coronary blood flow in humans (Akhmedov & Marín‐García, 2013; Laflamme & Murry, 2011; Miyagawa et al., 2002; Urbanek et al., 2005).

In addition to the classic rodent models in regenerative medicine, there has been much focus on the impressive ability for cardiac regeneration in fish and amphibians, particularly zebrafish and axolotl (Bise et al., 2020; Cano‐Martínez et al., 2010; de Preux Charles et al., 2016; Marro et al., 2016; Voss et al., 2009). These studies have revealed activation of ubiquitous and important intracellular signalling pathways, but have also shown that part of the regeneration ability of fish and amphibians is due to the lower metabolism of these ectothermic hearts (Ausoni & Sartore, 2009; Dittrich et al., 2020; Price et al., 2019). It is also likely that the relatively low blood pressures in fish and amphibians provide for higher tolerance to cardiac insult (Jewhurst & McLaughlin, 2016). Therefore, we surmised that it may be valuable to investigate the possibility of a reptilian model for cardiac regeneration, because the blood pressure of reptiles is significantly higher and hence closer to the mammalian state (Jensen & Christoffels, 2020). Typical lizard systolic blood pressure ranges between 40 and 60 mmHg, but it is highly variable across species (Schulte et al., 2015). Nevertheless, the axolotl, which is commonly used in cardiac regeneration research, has a systolic blood pressure of only 20–30 mmHg (Meyer et al., 2022).

Generally, reptiles are phylogenetically and metabolically closer than fish or amphibians to mammals including humans (Cherlin, 2024; Modesto & Anderson, 2004) and therefore valuable for finding conserved markers and factors for potential healing abilities after myocardial infarction or cardiac injury. Numerous examples of regenerative processes have been described in squamate reptiles, including tail and limb regeneration (Alibardi, 2009; Jacyniak et al., 2017), brain (Austin et al., 2023; López‐García et al., 1992) and cardiac regeneration (Jacyniak & Vickaryous, 2018). Relatively, little is known about the processes underlying the myocardial regeneration induced through the experimental myocardial necrosis in reptiles. The reptilian myocardium consist of the inner spongious and the distinct outer compact layer (Gregorovicova, 2024; Jensen & Christoffels, 2020) with good oxygen supply from the coronary arteries (Hagensen et al., 2008; Ostadal et al., 1999).

The leopard gecko (*Eublepharis macularius*) is an excellent reptilian model for studying regeneration with tremendous healing potential during its entire lifespan (Hoekstra et al., 2020; Hutchins et al., 2016; Tollis et al., 2015). It has a closer phylogenetic position to mammals than the teleost fish (Hedges, 2009) and is easily maintained in laboratories. The present study fills the knowledge gap between traditional models of myocardial healing response in poikilothermic (zebrafish, axolotl) (Cano‐Martínez et al., 2010; González‐Rosa et al., 2017; Jopling et al., 2010) and homeothermic (neonatal mice, rats) (Lam & Sadek, 2018; Wang et al., 2020) vertebrates. Such an approach could lead to uncovering the traits that are common and present for all vertebrates (ancestral traits) (Cutie & Huang, 2021; Jaźwińska & Sallin, 2016; Vivien et al., 2016).

## 2 METHODS

### 2.1 Ethical approval

All animals were bought as captive bred from certified sellers of exotic animals in the Czech Republic, reared and transported under conditions specified in the Czech republic animal act no. 246/1992 coll. on the protection of animals against cruelty. All experiments were performed under institutional ethics committee approval 8615/2019‐MZE‐17214, license no. CZ 02470. The investigators understand the ethical principles under which the journal operates and their work complies with the journal's animal ethics checklist.

### 2.2 Animals

Twenty‐eight adult female leopard geckos (*Eublepharis macularius*; 30–60 g and over 9 months of age) were kept separately in glass vivaria (dimensions of 20 × 40 × 20 cm) at the Institute of Anatomy, First Faculty of Medicine, Charles University and in the Department of Zoophysiology, Aarhus University at 28°C, 50–70% humidity, and light–dark cycle of 12:12 h. Animals were monitored on a daily basis, and they did not see each other in order to minimize their stress. The same sex guaranteed homogeneity in terms of sufficient *n* for statistics. The animals were habituated for at least 1 month before the experiments started. They were fed once a week with crickets supplemented by calcium and vitamins and they were provided with water ad libitum. Animals were randomly divided into five groups according to experimental design: control group and four healing interval groups (7, 14, 21 and 28 days after cryoinjury [dac]). Animals in the control group were euthanized without cryoinjury after 1 month of habituation to the environment. Animals from the healing interval groups were habituated at least for 1 month to the environment. After habituation, animals were successively operated, and fed 3 days after surgery. With the exception of the 7‐day healing interval, animals in the other healing intervals were fed on a weekly basis. After specific healing intervals, animals were anaesthetized by isoflurane and alfaxan multidose 10 mg/mL (0.5 mL per animal), and sacrificed using an overdose of Exagon (pentobarbital, 400 mg/mL, 0.5 mL per animal) in accordance with the animal protection legislation of the Czech Republic (accreditation no. 8615/2019‐MZE‐17214, license no. CZ 02470).

### 2.3 Surgical procedure of cryoinjury

The cryoinjury was performed under general anaesthesia (isoflurane inhalation) following standard protocols (Sladky & Mans, 2012). The anesthetized lizard was positioned on the heat pad (28°C) and intubated for ventilation with 4–5% isoflurane for 5–15 min with subsequent reduction to 1–2.5%. The procedure normally lasted around 30 min. Analgesia (0.2 mg/kg Meloxicam: Metacam inj. a.u.v., BASF Ingelheim am Rhein, Germany) was administered i.m. in the right front leg under general anaesthesia. The anaesthesia did not exceed 60 min. The depth of anaesthesia was checked by pressing the tail and forelimb with tweezers. The chest in the fifth intercostal area on the animal's left side was opened to gain access to the pericardium. Cryoinjury was inflicted by spraying nitrous oxide (N_2_O) at −89°C using a cryogenic tool CryoPen^®^ M (H&O Equipments Ath, Belgium) with the micro‐applicator of 1–3 mm lesion diameter (micro‐applicator marked by blue/red colour dots) on the ventricular apex in one click (0.5 s). After quick freeze absorption into the myocardium, the animal was stitched with individual sutures (using surgical non‐resorbing material, Marpolen blau 6/0 2× DRT 12 Catgut GmbH, Markneukirchen, Germany). The ventricular apex was chosen because of its importance for the direction of heart contraction (apex‐to‐base pattern), which is similar in all vertebrates (Jensen et al., 2012). Moreover, the ventricular apex represents an easy‐to‐find location with low cryoinjured variability. The isoflurane was discontinued, and the endotracheal cannula was removed when spontaneous ventilation resumed. The animal was then monitored for the next 2 h while fully recovering from anaesthesia. The animal did not receive additional analgesics after awakening from anaesthesia due to potential interference with the experimental design. Animals were fed for the last time 1 week before surgery and the first feeding started 3 days after surgery. All animals exhibited normal prey‐catching behaviour and they were fed after 3 dac. This interval was chosen due to completion of metabolic clearance of the anaesthetics in geckos to prevent them from vomiting the food after anaesthesia.

Five untreated lizards were sampled as controls (*n* = 5). The specific sampling intervals of the healing period were 7 (*n* = 6), 14 (*n* = 6), 21 (*n* = 6) and 28 (*n* = 5) dac (Table 1). Heart sampling was started by optical mapping and followed with morphological processing. Animals were weighed before cryoinjury and after the specific healing interval just before harvesting the heart to obtain information about potential weight loss/gain during the particular healing period. In the case of control lizards, the animals were weighed 1 week before the experiment and on the day of the sampling.

**TABLE 1:** Numbers of animals used in experiments.

	Optical mapping	MicroCT	Histology and IHC
Total	28	10	10
Control	5	1	2
7 dac	6	2	2
14 dac	6	2	2
21 dac	6	3	2
28 dac	5	2	2

Abbreviation: dac, days after cryoinjury.

### 2.4 Optical mapping

In vitro optical mapping was performed using Ringer solution without lactate at 28°C (Jensen et al., 2012; Kvasilova et al., 2020). The heart was excised from the chest and the pericardium was removed. Then, the voltage sensitive dye di‐4‐ANNEPS (Biota, cat. no. 61010 Biotium Inc, Fremont, CA, USA) at a concentration of 60 µL stock (1.25 mg/mL in dimethylsulfoxide) in 1 mL Ringer solution was applied for 10 min on ice. After this staining period, the heart was rinsed with ice‐cold Ringer solution and pinned down in a silicone‐lined dish and mapped using an ULTIMA L camera and THT mesoscope with tandem Leica optics (both devices from Brain Vision, Tokyo, Japan). The hearts were additionally warmed during the optical mapping in the bath to 28°C by a heating stage, which was positioned under the bath. The hearts were not retrogradely perfused. Instead, a bath with a high level of oxygen was used, and therefore the hearts were not hypoxic. Moreover, the hearts showed good long‐term stability in the culture with bubbling of oxygen alone. The system was equipped with a LEX3 LED light source (Brain Vision) providing high intensity and high stability illumination (Olejnickova & Sedmera, 2020). The signal spreading activity was recorded from both sides of the heart (the ventral part as well as the dorsal part) at a temporal resolution of 4, 8 and 16 ms (all analysed). Epicardial activation maps were generated, and ventricular epicardial activation time pattern, heart rate and atrioventricular delay (AVd) were analysed by BV_Ana software (SciMedia, Brain Vision). AVd was chosen for its universality of spreading signal for orchestrated and coordinated ventricular contraction, because squamates do not have a His–Purkinje axis for signal spreading across ventricle (Gregorovicova et al., 2018). The AV interval is strongly influenced by the autonomic nerve tone, and we showed previously in poikilotherms (Olejnickova et al., 2021) that it is a meaningful parameter to measure. In this case, we believe the observed AV interval shortening reflects general sympathetic system activation.

After optical mapping, the hearts were fixed overnight in 4% paraformaldehyde for 24 h at 4°C, rinsed twice with phosphate‐buffered saline (PBS), and stored in 70% ethanol at 4°C or fixed directly in Dent's fixative (20% dimethyl sulfoxide in methanol) for 24 h at 4°C. The hearts were randomly divided into two groups and processed for either micro‐computed tomography (microCT; *n* = 10) or histological examination (*n* = 10) after such fixation (Table 1).

### 2.5 MicroCT

Hearts from nine individuals were micro‐CT‐scanned using SkyScan 1272 (Bruker micro‐CT, Kontich, Belgium). Prior to scanning, specimens were X‐ray contrasted by phosphotungstic acid according to Metscher (2009). Scanning parameters were set depending on the specimen's size and X‐ray density of the contrasted tissue. Pixel size was set between 10 and 20 µm, source voltage 60–90 kV, source current 111–166 mA, rotation step 0.4, filter Al 1 mm or Al 0.5 mm + Cu 0.038 mm, 180° rotation. Projection images were reconstructed using NRecon software (Bruker). Cross‐sectional images were used for 2D and 3D visualizations (DataViewer, CTVox; Bruker).

### 2.6 Histology and immunohistochemistry

After optical mapping, the isolated hearts were first documented by macrophotography using an Olympus DP71 camera fitted on an Olympus SZX12 dissecting microscope with incident illumination and then processed for paraffin embedding. The blocks were serially sectioned at 8 µm in the frontal plane for further histological and immunohistochemical analysis. Morphological changes in trabecular organization, scar formation, restored tissue and levels of fibrous tissue in the hearts were assessed by histological staining. Specifically, Alcian blue with haematoxylin and eosin staining was used for overview description, and Picrosirius Red (PSR) standard collagen staining for fibrosis characterization. To address the changes in myocardial differentiation, immunohistochemical staining for a myocardial marker (myosin heavy chain; MF20, DSHB Iowa, IA, USA, cat. no. AB2147781104; Helsby et al., 2014) was used following a previously used protocol (Olejnickova et al., 2021). To detect cardiac cell proliferation, proliferating cell nuclear antigen (PCNA) was used with detection in transmitted light or in fluorscent light. PCNA (Dako M0879 Santa Clara, CA, USA) at dilution 1:100 was applied overnight, and then, after three rinses with PBS, goat anti‐mouse Cy5 (JIRL West Grove, PA, USA, 115‐175‐146) as a secondary antibody at dilution 1:200 was applied for 90 min, followed by wheat germ agglutinin (WGA; Thermo Fisher Scientific, W11261 Waltham, MA, USA) at dilution 1:50 applied for 60 min, and then 4′,6‐diamidin‐2‐phenylindol (DAPI; BioVision B1098 abcam, Cambridge, Great Britain; dilution 1:1000 applied for 30 min) counterstaining was used for the observation in in fluroescent light.

Sections were mounted and analysed in transmitted light using an Olympus BX51 microscope fitted with an Olympus DP80 CCD camera, and with confocal microscopy (Olympus BX61 with FluoView system and Leica Stellaris 5) for the fluorescent staining. Analyses of the histological and immunohistochemical stainings were performed with ImageJ software (NIH, Bethesda, MD, USA) with a focus on the fibrotic area (cross‐sectional area) percentage in bright field as well as in polarized light with a ×40 objective (Sedmera et al., 2024) on three sections across the whole injured area of the left ventricular part (cryoinjured area at 7, 14, 21, 28 dac and healthy area in the control) per animal with PSR staining. The same method was used for the cardiomyocyte amount: cross‐sectional area in visible light with a ×40 objective for PSR staining, analysed with ImageJ software. Boundaries of the cryoinjured area were defined according to PSR and Alcian blue with haematoxylin and eosin staining, more specifically by staining concentration in the left part of the ventricle.

### 2.7 Statistical analyses

Statistical analysis of optical mapping followed a linear model with a robust version of heteroskedasticity according to Hothorn et al. (2008) in the R program with the multcomp package, with statistical level of significance *P *< 0.05. The graphs were computed in GraphPad Prism version 8.0.2 (GraphPad Software Inc., San Diego, CA, USA). One‐way ANOVA was used for PSR analyses with statistical level of significance *P *< 0.05. The weights were analysed in Prism version 8.0.2. as percentage differences before cryoinjury and after a particular healing interval before harvesting the heart by Student's *t*‐test. Values are reported as means ± SD.

## 3 RESULTS

**FIGURE 1:**
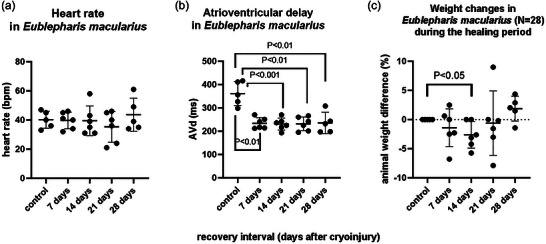
Longitudinal characterization of changes after cryoinjury in *Eublepharis macularius* (*n* = 28). (a) In vitro heart rate during the healing period. (b) Atrioventricular delay. (c) Changes in body mass. Cryoinjury had no significant impact on heart rate (a) over the 28 days of healing. The fastest atrioventricular conduction from the healing period intervals occurred at 14 dac (b). The cryoinjury caused a significant decline in body mass at 14 days after injury of 1.5 g (2.6% of their starting mass; c). Positive values indicate gaining weight (%), negative values losing weight (%). Values are presented as means ± SD.

The baseline in vitro heart rate during the optical mapping was 40 ± 9 beats per minute (bpm) (Figure 1a) with a corresponding AVd of 250 ± 51 ms. The heart rate was 40 ± 6 bpm in the control hearts with a longer AVd (average = 362 ± 52 ms) (Figure 1b). The fastest atrioventricular conduction from the healing period intervals was observed in 14 dac (*P* = 0.0009). Cryoinjury had no significant impact on heart rate (*P* = 0.851) or ventricular epicardial activation time (*P* = 0.891) over the 28 days of healing. There were differences between controls (*n* = 5) and the particular recovery interval in AVd during the healing period (*P* = 0.002). The cryoinjury caused a significant (*P* = 0.048) decline in body mass at 14 days after injury of (1.5 g, 2.6% of their starting mass, Figure 1c).

**FIGURE 2:**
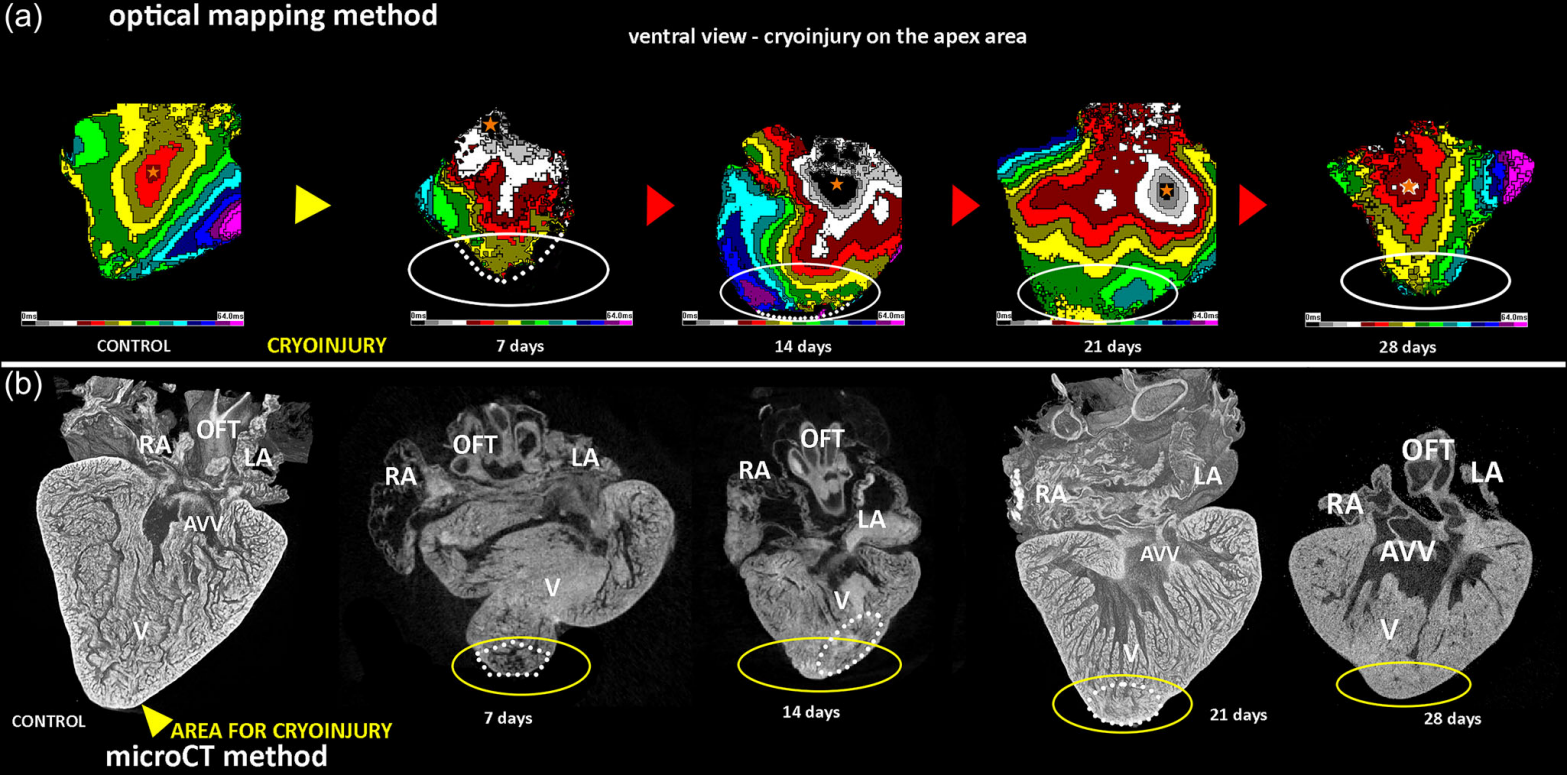
Time course of cryoinjury resolution. (a) Optical mapping of adult female *Eublepharis macularius* hearts. A series of ventral views with the cryoinjury inflicted on the apex area (white ellipse). The hearts at 7 and 28 days after cryoinjury (dac) were mapped under the same magnification as the rest hearts but the animals and therefore their hearts were smaller. Map area 1 × 1 cm. Orange asterisks indicate the first activated epicardial area; the orange arrows show direction of signal spreading over the epicardial surface of the ventricle. The activation maps are expressed as isochronal contour lines with each colour band corresponding to 4 ms interval; white dots indicate missing signal from the tissue in the cryoinjured apex area. Optical mapping time course differed during the healing period at 28 dac in signal spreading over the ventricle. The signal spread from the base of the heart towards the apex, and there was no optical signal over the cryoinjured area in the apex at 7 and 14 dac. There was a functional recovery of signal spreading over the whole ventricular surface by 21 and 28 days after injury. (b) MicroCT of adult *Eublepharis macularius* hearts over the timeline during the healing period. The microCT examination showed regeneration of cryoinjured tissue within 21 and 28 days consistent with the results of the optical mapping and histology. Yellow ellipse, apex area; white dots (polygon and ellipses), cryoinjured area. AVV, atrioventricular valve; LA, left atrium, OFT, outflow tract; RA, right atrium; V, ventricle.

Moreover, an optical mapping time course showed differences during the healing period of 28 dac in signal spreading over the ventricle. The signal spread from the base of the heart towards the apex, and there was no optical signal over the cryoinjured area on the apex in particular stages at 7 and 14 dac (black area, Figure 2a). However, there was a functional recovery of signal spreading over the whole ventricular surface by 21 and 28 days after injury. Consistent with this functional sign of recovery, the microCT examination revealed regeneration of cryoinjured tissue within 21 and 28 days (Figure 2b).

**FIGURE 3:**
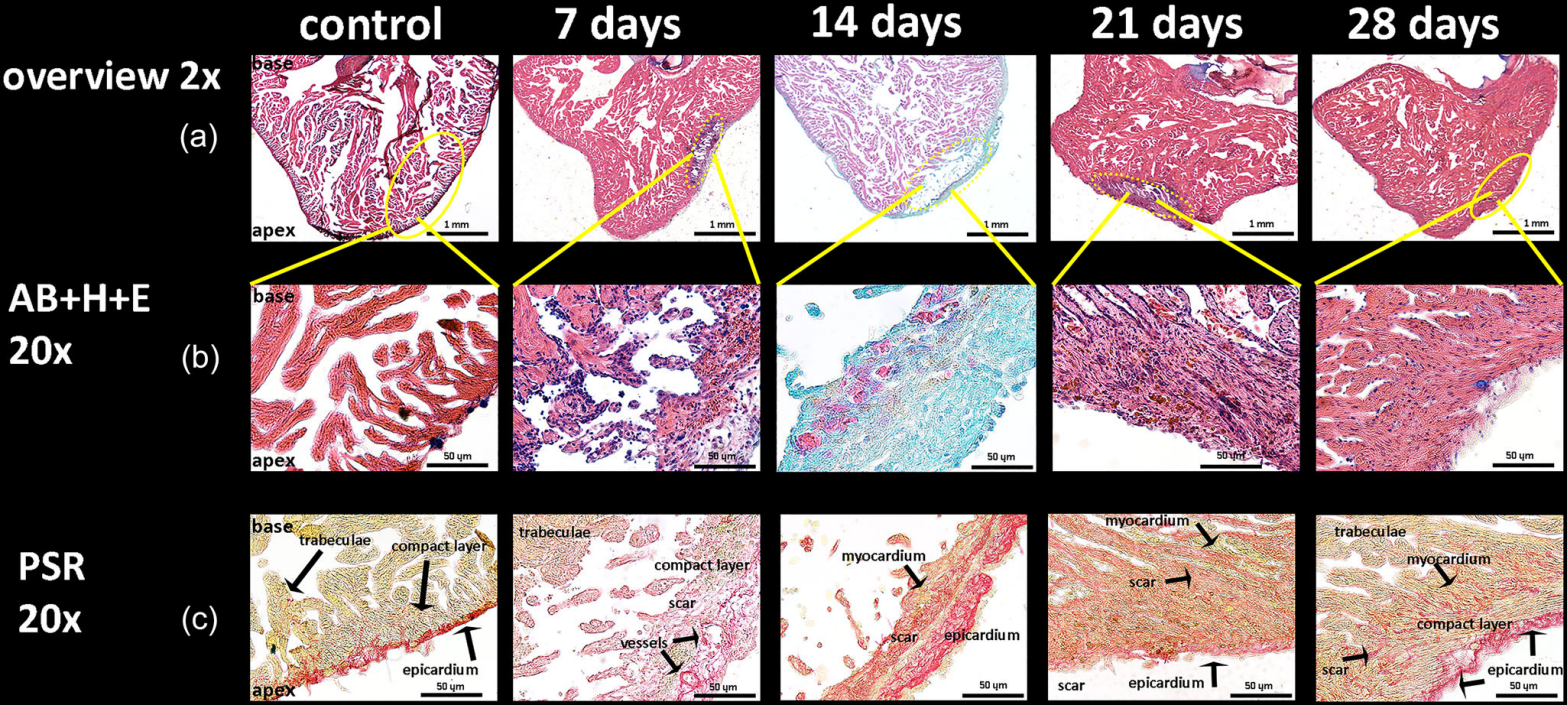
Histology and immunohistochemistry in timeline during the healing period in *Eublepharis macularius*. (a,b) Blue: Alcian blue, fibrotic tissue; pink: eosin, muscle; purple/brown: haematoxylin, nuclei. The overview staining by Alcian blue with haematoxylin and eosin showed large differences among stages in scar and fibrotic tissue formation. Separation and ballooning of the epicardium was observed at 7 dac together with epicardial activation. At 14 dac the necrosis and the degradation of the compact layer and trabeculae were more pronounced. At 21 dac the wound was smaller than at 14 dac and the scar was more compacted with a lot of new myocardial tissue and vessels. At 28 dac the scar was almost reabsorbed after 4 weeks of the healing period, but the thickening of the compact wall area was present. Yellow ellipse in control in (a): target area for cryoinjury; yellow dotted ellipses in (a): cryoinjured area. (c) Red: Picrosirius Red, collagen fibres (scar tissue); yellow: muscle. The fibrotic tissue was detected at all recovery intervals (red colour), but at 28 dac restoration of the trabeculae and compact layer similar to control was observed. The size of the cryoinjured area was approximately 5% of the total heart with the greatest extension of the injured area at 14 dac (approx. 8% of the total surface area). At 21 dac, the cryoinjured area was full of fibrotic tissue, but less than at 7 and 14 dac. At 28 dac, the injury extension was only 2% of the whole heart surface.

The healthy leopard gecko myocardium was mostly trabeculated but it also had a distinct compact layer (Figure 3). During the healing period the changes were observed in the cryoinjured tissue of the ventricular left part of the apex area in comparison to the healthy intact controls. The overview staining by Alcian blue with haematoxylin and eosin (AB/H+E) showed large differences among stages in scar and fibrotic tissue formation (Figure 3a,b). Specifically, separation and ballooning of the epicardium was observed at 7 dac together with signs of epicardial activation (e.g., thickening of the epicardium, increased subepicardial space, increased extracellular matrix). At 14 dac the necrosis and degradation of the compact layer and trabeculae were more pronounced. The space between the inner (trabecular) myocardium and the outer myocardial layer, which lay subepicardially, was infused with blood. The scar had a larger diameter than at 7 dac but there was also observed formation of the new vessels and myocardium. Numerous coronary vessels were observed and they localised in the cryoinjured area in the activated subepicardium. After 21 dac, the closing wound was observed as shrinking of the wound area in comparison with 14 dac. At 28 dac the scar was almost reabsorbed after 4 weeks of healing, but a thickening of the compact wall area was present. The trabeculae and compact layer were regenerated, which was apparent also from the PSR staining showing resorption of the excess collagen (Figure 3c).

**FIGURE 4:**
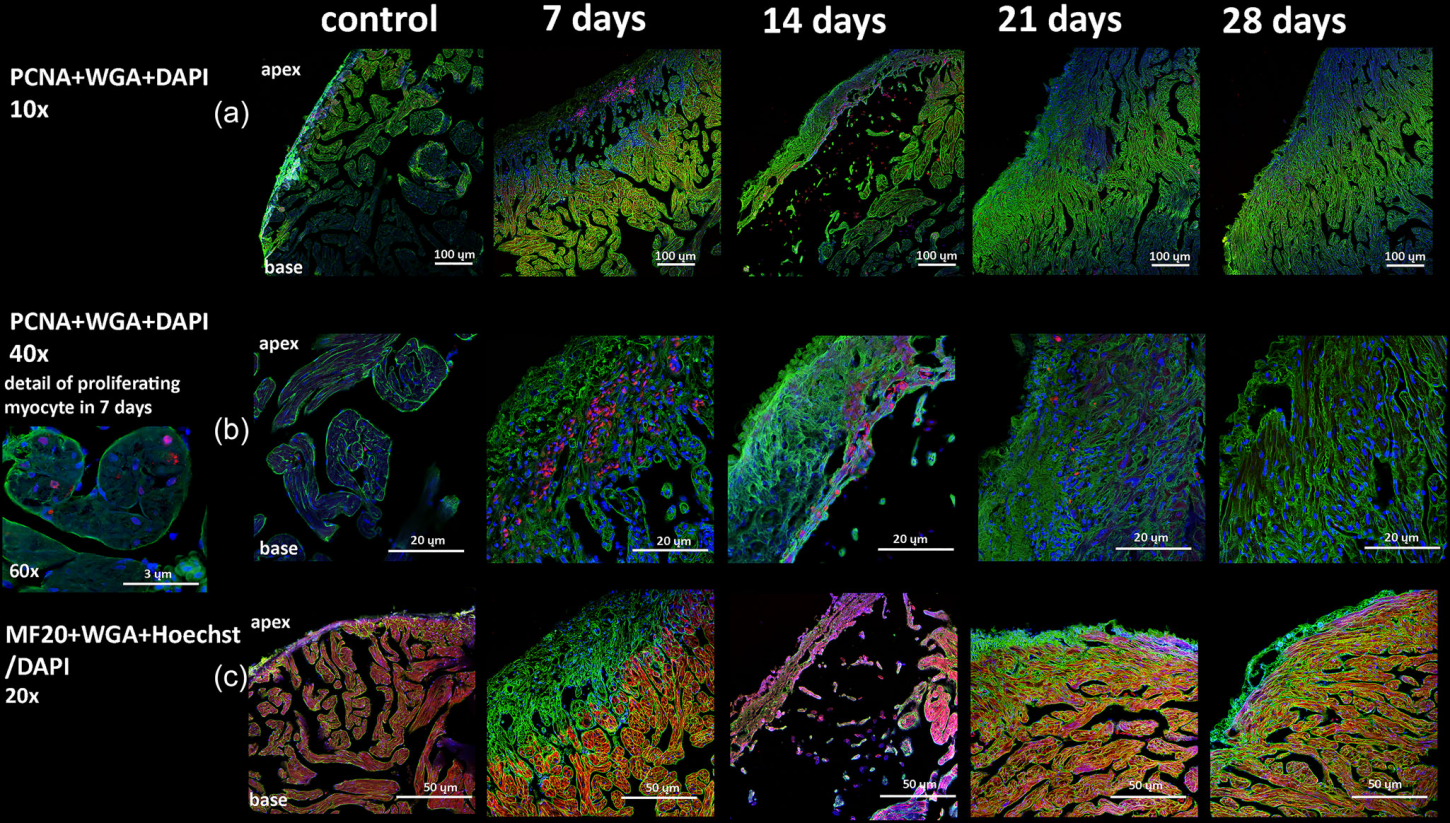
Immunohistochemistry in timeline during the healing period in *Eublepharis macularius*. (a, b) PCNA in the fluorescent light. Purple: positivity for PCNA; green: membranes; blue: nuclei. PCNA was clearly detected and observed in the cryoinjured area during the healing period (a, b). The highest amount of actively proliferating cells was observed at 7 and 21 dac (which is illustrated by a graph in Figure 5). At 14 dac degradation of the tissue was observed, so the amount of proliferating cells was lower than at the stages previously mentioned. At 28 dac there were almost no proliferating cells present in the cryoinjury site and the tissue was indistinguishable from control. (c) Red: MF20, muscle; green: WGA, fibrotic tissue (scar) and new membranes; blue: Hoechst/DAPI, nuclei. MF20 staining showed clearly observed muscle and membranes degradation at 7 dac, which pointed to necrosis. The restoration of the trabeculae as well as new cardiomyocytes budding from the subepicardial myocardium was observed at 14 dac. At 21 dac the epicardium and subepicardium were still activated. The new trabeculae and compact layer organization showed good differentiation of these two myocardial compartments. The histological appearance of the scar area at 28 dac showed that it was restored to a state similar to controls (healthy animals) with the only observed difference being the thickening of the tissue in the compact layer as a residuum after injury as well as the scar residua. Objects with bright fluorescence in all wavelengths are erythrocytes with nuclei.

**FIGURE 5:**
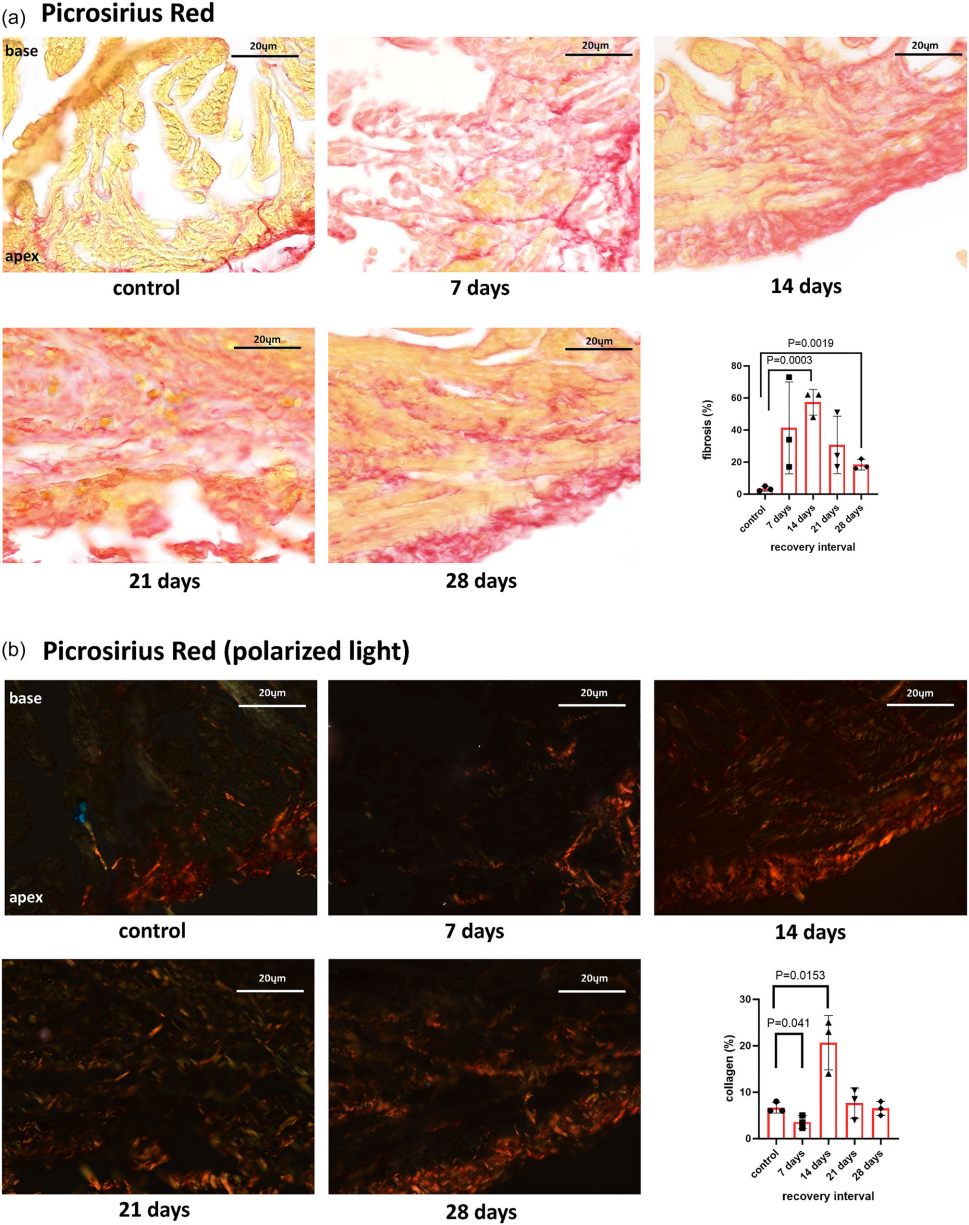
Amount of fibrosis (a) and collagen (b) in cryoinjured tissue in *Eublepharis macularius*. (a) Fibrotic tissue in the scar was the most extensive at 14 dac. There was a reduction of fibrosis at 28 dac relative to the control. (b) The amount of collagen was significantly lower at 7 dac and higher at 14 dac in comparison to controls. Hearts, *n* = 10; sections, *n* = 3 per heart. Picrosirius Red staining. Magnification ×40. Values are means ± SD. Red: fibrotic tissue; red colour fibrotic tissue; green colour muscle; yellow colour ‐ muscle.

PCNA, expressed in the nuclei of S‐phase cells and used as a cell proliferation marker (Sedmera & Thompson, 2011), was evaluated in fluorescent as well as transmitted light. PCNA was clearly detected and observed in the cryoinjured area during the healing period (Figure 4a,b). The highest number of actively proliferating cells was observed at 7 and 21 dac (Figure 5). At 14 dac degradation of the tissue was observed when the necrosis after cryoinjury was at its highest stage but also with the start of budding new tissue as well. Therefore, the number of proliferating cells was smaller than at the stages previously mentioned. At 28 dac there were almost no proliferating cells present in the cryoinjury site and the tissue was almost indistinguishable from control. In recapitulation, the highest number of proliferating cells was observed at 7 dac and 21 dac. At 14 dac, the number of proliferating myocytes was the lowest. At 28 dac, the number dropped almost to that of the control state.

To address the changes in myocardial differentiation, immunohistochemical staining for the myocardial marker myosin heavy chain (MF20) was applied (Figure 4c). MF20 staining and counterstaining with wheat germ agglutinin (WGA), which marks the outer and inner cell membranes, revealed regenerative process of the myocardial trabeculae and the compact layer. At 7 dac, there was clear observation of muscle necrosis and membranes degradation. At 14 dac, the restoration of the trabeculaewas seen as well as new cardiomyocytes sprouted from the subepicardial myocardium. The space between the epicardium and myocardium was infused by blood. At 21 dac, the epicardium and subepicardium were still active. The ballooning between the myocardium and epicardium receded a little. The new trabeculae and compact layer organization showed good differentiation of these two myocardial compartments (Figure 4c). The histological appearance of the scar area at 28 dac was similar to controls (healthy animals), but the tissue of the compact layer was thicker as a residuum after injury as well as the scar residua.

PSR was used to detect fibrosis in the cryoinjured tissue (Figure 5), which significantly differed across the stages (*P* = 0.015). Fibrotic tissue in the scar was the most extensive at 14 dac (*P* = 0.0003; Figure 5a). Fibrosis in the damaged tissue increased at 14 dac (17 times more than in control) and then gradually decreased during the healing period. There was a reduction of fibrosis to 5.5 times greater than in control at 28 dac (*P* = 0.0019; Figure 5a). The amount of collagen was significantly lower at 7 dac (*P* = 0.04) and higher at 14 dac in comparison to controls (*P* = 0.015; Figure 5b). In the case of cardiomyocyte amount, there was a significant difference among stages during healing period (*P* = 0.0018, data not shown) with the greatest difference in cardiomyocyte amount between control and 7 dac (*P* = 0.0074). Such an amount had approximately six time fewer cardiomyocytes at 7 dac than in control. There was no difference in cardiomyocyte amount between control and 28 dac. The cryoinjured area size was approximately 5% of the total heart with the greatest extension of the injured area at 14 dac (approx. 8% of the total surface area). At 28 dac, the injury extension was only 2% of the whole heart surface.

## 4 DISCUSSION

Our study demonstrates that the heart of the leopard gecko (*Eublepharis macularius*) regenerates within weeks after cryoinjury. These findings align well with the reported tremendous healing potential of the brain and the tail in this species (Austin et al., 2023; Jacyniak et al., 2017). Moreover, cardiomyocytes constitutively proliferate in the postnatal leopard gecko heart (Jacyniak & Vickaryous, 2018), which is important for cardiac healing after injury in reptiles.

Myocardial regeneration has been intensively studied (Kikuchi & Poss, 2012; Laflamme & Murry, 2011), and the large regenerative capacity of some species of fish and amphibians offers interesting animal models to study the underlying cellular and molecular processes (Jewhurst & McLaughlin, 2016). These species, however, have rather low blood pressures that may confer extraordinarily high tolerance to impaired cardiovascular function and extra protection in the recovery period (González‐Rosa et al., 2017). We therefore considered it imperative to enquire whether reptiles with their higher blood pressures and more compact ventricles also possess a regenerative capacity. Hence, reptiles are important models not only for evolutionary closeness to mammals (Hedges, 2009) but also for their well‐developed distinct trabeculae and compact layer (Gregorovicova et al., 2022), higher blood pressure (Galli et al., 2006) and metabolic rate (Kikuchi & Poss, 2012). Thus, their hearts are more akin to the mammalian state than hearts of fish and amphibians. Therefore, we chose a reptilian model species—the leopard gecko (*Eublepharis macularius*; Agarwal et al., 2022) for this study, because of its great regenerative abilities and easy‐to‐care‐for qualities.

The regeneration of the leopard gecko heart after ventricular cryoinjury starts with epicardial ballooning, and the loss of myocardial viability was observed. The outer myocardium, lying subepicardially, was involved in the restoration of trabeculae and compact myocardium similar to results reported in newt (Piatkowski et al., 2013). New myocardium was fully viable and functional (Figure 2) after a 4‐week healing period with almost no discernible scar presence.

The critical question is how fibrosis is developed (Furtado et al., 2016). Cardiac fibrosis development as well as scar formation and resorption are not yet described in squamate reptiles. Fibrosis results from processes that include oxidative stress or inflammation, which occur also after cardiac injury (Castillo‐Casas et al., 2023a, 2023b; Tsutsui et al., 2011). Thus, this study is focused also on the temporal aspects of scar development and resorption. In addition, the fibrous tissue presence in the epicardial layer, which was observed also in the control heart, is standard and a healthy condition, not only in embryos (Quijada et al., 2020) but also found in postnatal vertebrate hearts (Uscategui Calderon et al., 2023). The more important question lies not in the presence of the subepicardial fibrous tissue (also connected with the pericardium) but its transmural expansion in the ventricular muscle in the injured area (Quijada et al., 2019) as we observed in the injured hearts. The next step in reptilian cardiac healing is scar remodelling, where the extracellular matrix is degraded and is substituted by myocardial tissue with adequate vascular supply. These observations align with the successful orchestration of the immune response that together with the regenerative processes in cardiac tissue involves a complex interplay (Lafuse et al., 2020). This should be a future research direction. The leopard gecko shows a similar myocardial tissue structure and architecture to controls after a 4‐week period. Moreover, lizards show almost no fibrotic scar after the whole healing period. These findings are in agreement with findings in zebrafish, where scar tissue disappear swithin 60 dac (Bise et al., 2020), but it seems that geckos healed faster than zebrafish. This may be due to their higher metabolism and body temperature of the geckos and points to lizards as an example of adult amniotic vertebrates being able to heal without fibrotic scar as described for neonatal mice or rats (Lam & Sadek, 2018; Wang et al., 2020). Moreover, we predict that after a 4‐week regenerative period, the fibrosis would drop to the control state.

It is well known that the adult cardiac muscle in mammals does not heal without a permanent fibrotic scar (Weinberger & Riley, 2024). Here, adult leopard gecko females heal their cryoinjured ventricles. Moreover, it was demonstrated that gecko cardiomyocytes proliferated even in adulthood (Jacyniak et al., 2025). In our work, observations of high proliferative activity of the cardiomyocytes in the vicinity of the cryoinjured area were also made. We show that the intrinsic proliferative ability of the reptilian cardiomyocytes forms the basis of regenerative myocardial tissue restoration after injury.

In cryoinjured leopard gecko the AVd was shorter than in controls. A short AVd is typical for birds and mammals but not for reptiles (Anderson et al., 2018), which typically have a much lower heart rate. Such an observation could lead to a different response to stress reaction (Olejnickova et al., 2021) or to cardiac arrhythmia (Chi et al., 2008). AVd is defined as the time between atrial and ventricular activation (Dreifus et al., 1971). Thus, shorter AVd allows faster signal spreading between the atria and ventricle and therefore it could be linked to stress reaction with increased adrenergic tone and positive dromotropic effect (Olejnickova et al., 2021). Metabolism and its rate are other important factors in cardiac regeneration (Gut et al., 2017; Kikuchi et al., 2012; Tzahor & Poss, 2017). However, little is known about such processes in squamates. Moreover, males and females could differ in hormonal–metabolic impact on the healing potential during cardiac regeneration. Hormonal–metabolic impact on the healing potential between males and females in geckos is generally reported (Khaire et al., 2021; Li et al., 2025).

Revealing the mechanisms of myocardial healing via squamate species models is important from the evo–devo point of view. Moreover, it is also a basic evolutionary question in itself that sheds light on the general ability of tissues to restore function after injury (Castillo‐Casas et al., 2023a; Huang et al., 2024). In fact, the squamates are bridge species and should be used for a better understanding of cardiac healing processes with possible knowledge translation to mammals as they are closer relatives than zebrafish or amphibians (Hedges, 2009). Such research could better precise the reveal the cardiac block, which prevents regeneration of damaged myocardium in adult mammals.

In conclusion, leopard geckos heal myocardial damage within 4 weeks and restore ventricular structure and function, and our histological analysis suggests the formation of new myocardium, with PCNA staining revealing more cycling nuclei. The leopard gecko provides an amniote model to understand cardiac regeneration without replacement fibrosis, and may be able to provide important novel insights into myocardial healing across the vertebrate lineages and bridge the gap between non‐ischaemic regenerative and ischaemic reparative processes during cardiac regeneration.

### 4.1 Study limitations

The study limitation lies in the reliance on the *ex vivo* monitored heart, which means there is no connection to the autonomic nervous system and other vital systems as in the intact organism. Moreover, cryoinjury has a different damage process than coronary occlusion, which is the next step for subsequent research together with extending the observations after 4 weeks of cardiac injury. Another study limitation could lie in the sex of the leopard geckos used. We used only females due to the fact that males and females could differ in hormonal–metabolic impact on healing potential during cardiac regeneration. Therefore, we did experiments only on females to reduce the potential hormonal–metabolic variability possibly involved in cardiac regeneration.

## AUTHOR CONTRIBUTIONS

Martina Gregorovicova: conceptualization; data curation; formal analysis; investigation; manuscript editing; methodology; project administration; supervision; validation; visualization; writing the original draft. Barbora Sankova: investigation; validation. Martin Bartos: methodology; validation; visualization. Bjarke Jensen: manuscript editing. Tobias Wang: manuscript editing. David Sedmera: funding acquisition, manuscript editing. All authors have read and approved the final version of this manuscript and agree to be accountable for all aspects of the work in ensuring that questions related to the accuracy or integrity of any part of the work are appropriately investigated and resolved. All persons designated as authors qualify for authorship, and all those who qualify for authorship are listed.

## CONFLICT OF INTEREST

The authors declare that they have no conflicts of interest.

## Data Availability

All data is available as Supporting information upon request to corresponding author.
